# A meta-analytic evaluation of the correlation between event-free survival and overall survival in randomized controlled trials of newly diagnosed Ewing sarcoma

**DOI:** 10.1186/s12885-020-06871-9

**Published:** 2020-05-05

**Authors:** Kazuhiro Tanaka, Masanori Kawano, Tatsuya Iwasaki, Ichiro Itonaga, Hiroshi Tsumura

**Affiliations:** Department of Orthopaedic Surgery, Faculty of Medicine, Oita University, 1-1 Idaigaoka, Hasama, Yufu, Oita 879-5593 Japan

**Keywords:** Ewing sarcoma, Randomized controlled trial, Chemotherapy, Endpoint, Surrogacy, Overall survival, Event-free survival

## Abstract

**Background:**

In randomized controlled trials (RCTs) of adjuvant treatment for malignant tumors, event-free survival (EFS) is considered the most acceptable surrogate for overall survival (OS). However, even though EFS has repeatedly been selected as a primary endpoint in RCTs of Ewing sarcoma (ES), the surrogacy of EFS for OS has not been investigated. This study aimed to evaluate the correlation between EFS and OS in RCTs of chemotherapy for newly diagnosed ES using a meta-analytic approach.

**Methods:**

We identified seven RCTs of newly diagnosed ES through a systematic review, and a meta-analysis was performed to evaluate the efficacy and adverse events associated with chemotherapy for previously untreated ES. The correlation between EFS and OS was investigated using weighted linear regression analysis and Spearman rank correlation coefficients (*ρ*). The strength of the correlation was evaluated using the coefficient of determination (R^2^).

**Results:**

A total of 3612 patients were randomly assigned to 17 treatment arms in the eligible RCTs. The meta-analysis revealed that the hazard ratios for OS and EFS showed significantly better results in the experimental treatment groups with increasing toxicities. The correlation between the hazard ratios for EFS and OS was good (R^2^ = 0.747, *ρ* = 0.683), and the correlation tended to be more favorable in cases of localized ES (R^2^ = 0.818, *ρ* = 0.929).

**Conclusions:**

Overall, the trial-level correlation between EFS and OS was good for newly diagnosed ES and was very good in cases of localized disease. EFS may be a useful endpoint in RCTs of ES chemotherapy, and it is worth verifying using individual patient data.

## Background

Ewing sarcoma (ES) is the second most frequent malignant bone tumor in children. However, it accounts for only 1% of all childhood cancers in the United States [1]. ES is a highly malignant disease involving the dense proliferation of round tumor cells without specific histologic differentiation. Tumors presenting a similar histological appearance also occur in soft tissues. However, such tumors are considered to be the same as ES arising in the bone because they present the same chromosomal abnormality and fusion gene [1]. Intensive multidrug chemotherapy and definitive local control are the standard treatment options for ES [2].

A standard regimen for newly diagnosed ES has been examined in some randomized controlled trials (RCTs). RCTs provide crucial evidence for determining standard treatments, and primary endpoints in RCTs are considered the most useful measures for judging the effectiveness of treatment options; this influences trial conclusion, and subsequently, patient survival. Overall survival (OS) is the preferred primary endpoint in RCTs, as it is an index that eliminates subjectivity and ambiguity because it can be clearly defined and judged. Additionally, because improvement in OS is the primary objective of cancer treatment, OS is an appropriate indicator for evaluating treatment efficacy. However, using OS as the primary endpoint in RCTs entails longer durations, larger sample sizes, and higher costs. In addition, multiple lines of new drugs may extend patients’ post-progression survival, making it difficult to assess the actual effects of the regimens used in the trial and the influence of post-protocol treatment. Event-free survival (EFS) has, therefore, been used as the primary endpoint in many RCTs of ES. Furthermore, EFS is an important measure that indicates the disease-free period in an adjuvant setting trial; it is a meaningful outcome in its own right, especially in RCTs of pediatric cancer treatment. However, the correlation between EFS and OS in RCTs of ES therapy has not yet been investigated.

Accordingly, in this study, data on all RCTs that involved chemotherapy for newly diagnosed ES were collected, and the correlation between EFS and OS was evaluated using a meta-analysis.

## Methods

### Study selection and data extraction

Based on the Preferred Reporting Items for Systematic Reviews and Meta-Analyses guidelines [3], we conducted a systematic search of PubMed, Scopus, EBSCOhost MEDLINE, and the Cochrane Central Register of Controlled Trials. We searched for all RCTs of ES published in English between January 1973 and October 2018. The study inclusion criterion was phase II or III RCTs of systemic chemotherapy for newly diagnosed ES without prior treatment. We excluded non-randomized clinical trials, reviews, and meta-analyses. RCTs retrieved by this search were screened independently by two authors and cross-checked (KT and MK).

We performed data extraction of the date of publication, trial name, patient accrual period, study phase, primary and secondary endpoints, regimens and doses used in the standard and experimental arms, number of patients, sex, age, number of metastatic cases, description of intention-to-treat (ITT) analysis, description of post-protocol treatment, radiological and histological responses to chemotherapy, survival data, and adverse events (AEs).

Data of medians, hazard ratios (HRs), 95% confidence intervals (CIs), and *P*-values were extracted for OS and EFS. The pathological response rate was defined as the proportion of assessed patients with > 90% tumor necrosis in the resected specimens. Data of 1-, 3-, and 5-year EFS and OS were extracted based on Kaplan-Meier estimates. If these data were not described, Kaplan-Meier curves of EFS or OS were used for estimation as binary proportions. Data were extracted and cross-checked by two authors (KT and MK). In cases of discrepancies between these two authors, other authors (TI or II) were consulted to reach a consensus.

### Statistical analysis

Pooled HRs and their corresponding 95% CIs were subjected to meta-analyses, and the values for EFS and OS were obtained. The odds ratios (ORs) and the corresponding 95% CIs for 1-, 3-, and 5-year EFS and OS were also calculated. Meta-analyses were performed using inverse-variance and a Mantel-Haenszel random- or fixed-effect model. The random-effect model was used when the *p*-value in the heterogeneity test was < 0.1. Heterogeneity was evaluated using Cochrane’s Q-test and I^2^ statistics. Meta-analyses were conducted using Review Manager software (version 5.3; Nordic Cochrane Centre, Cochrane Collaboration, Copenhagen, Denmark).

The association between EFS and OS was evaluated using a weighted linear regression test with the study sample size. The correlation between HRs for the surrogate endpoints and OS was assessed using Spearman’s rank correlation coefficients (ρ). The strength of the association was also investigated using the coefficient of determination (R^2^) [4, 5]. Coefficient values > 0.9 were defined as excellent, > 0.75 as very good, > 0.5 as good, > 0.25 as moderate, and ≤ 0.25 as poor [6]. Sensitivity analyses were performed to eliminate the treatment arms of high-risk and metastatic disease from the evaluation of surrogacy. Further sensitivity analyses were conducted by eliminating two old RCTs (IESS-I and -II).

Other statistical analyses were conducted using SAS (version 9.4; SAS Institute, Cary, NC, USA). *P*-values reflected two-sided tests, with *P* < 0.05 indicating statistical significance.

## Results

### Characteristics of RCTs included in the analysis

**Fig. 1:**
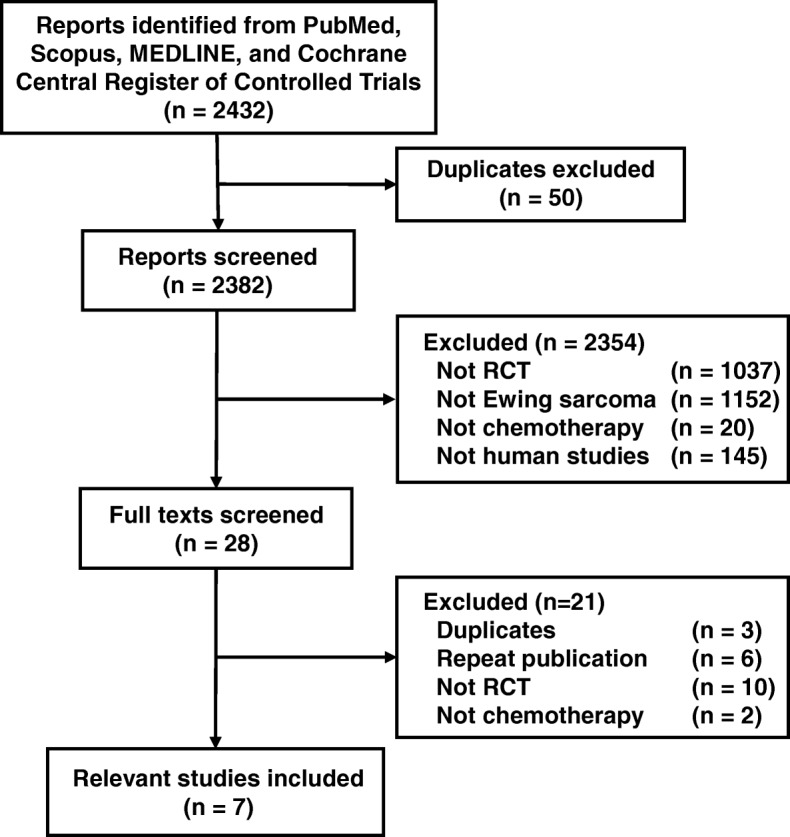
PRISMA flow diagram. PRISMA, Preferred Reporting Items for Systematic Reviews and Meta-Analyses; RCT, randomized controlled trial

In this systematic literature search, 2432 articles were identified. After excluding 50 duplicates, 2382 studies were further screened. The full texts of 28 articles were finally evaluated after excluding 2354 studies. Of the 28 studies, 3 duplicate publications, 6 repeat publications, 10 non-RCT studies, and 2 non-chemotherapeutic studies were excluded. The remaining 7 RCTs were considered eligible for the meta-analysis (Fig. 1). The characteristics of the eligible RCTs are summarized in Additional files 1: Table S1 [7–13].

In the 7 eligible RCTs, 3612 patients were randomly assigned to 17 treatment arms. All treatment arms comprised combination regimens with 3–5 cytotoxic drugs. One treatment arm included lung irradiation as the protocol treatment. No study included molecular-targeted therapy or immune therapy. Study phases and post-protocol treatments were not clearly described in any study, and ITT analyses were conducted in only 2. The primary endpoint was defined in 5 of 7 RCTs as EFS, including 3-year EFS, whereas 2 earlier studies described both survival time and time to relapse as major endpoints [7, 8]. All RCTs included both EFS and OS as efficacy measures of the trial. Whereas most RCTs focused on localized ES, 2 had subgroup arms for high-risk and metastatic disease, which included 277 patients. The mean of the studies’ median follow-up periods was 6.79 (5.1–8.5) years. Because the median EFS and OS were not reached in 7 and 5 treatment arms, respectively, analyses regarding median survival were not included in our study. The radiological response to chemotherapy was not described in any of the studies, and the histological response was assessed in only 2. Therefore, tumor responses could not be evaluated in the present study.

A significant difference in the HRs of EFS was observed between the control and experimental arms (HR 0.80, 95% CI 0.68–0.96, *P* = 0.01) (Additional File 2: Figure S1). Meta-analyses of the OS HRs revealed significantly better survival in the experimental arm than in the standard arm (HR 0.79, 95% CI 0.63–0.98, *P* = 0.03) (Additional File 3: Figure S2).

**Fig. 2:**
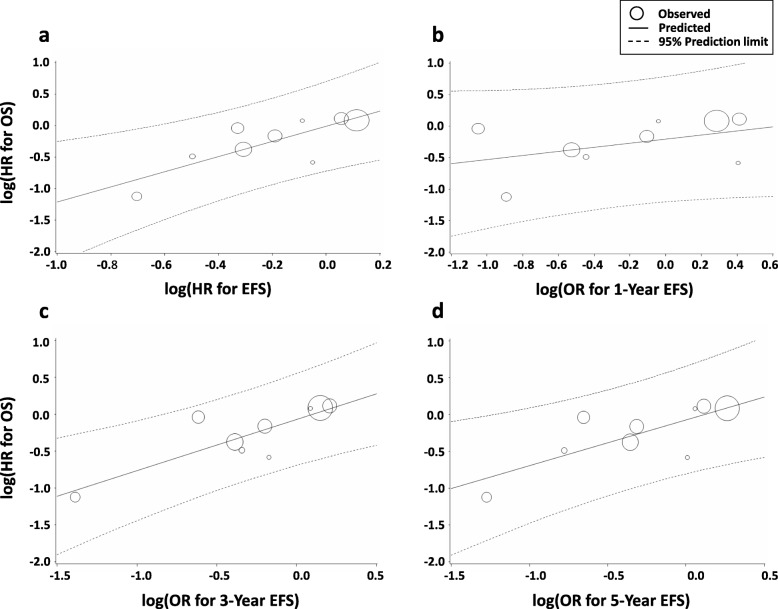
Correlation of EFS with OS HR for Ewing sarcoma. Correlation of (**a**) EFS HR, (**b**) 1-year EFS, (**c**) 3-year EFS, and (**d**) 5-year EFS. HR, hazard ratio; OR, odds ratio; OS, overall survival; EFS, event-free survival

Figure 2 shows forest plots for the treatment effects estimated by hazard ratios (HR) of the 2-year OS and 1-year PFS, TTP, and TTF for each trial.

### Correlations between EFS and OS

The trial-level correlation between the HRs for EFS and OS was good (R^2^ = 0.747, 95% CI 0.531–0.981) (Table 1, Fig. 2a). The Spearman’s rank correlation coefficient (ρ) was 0.683 (95% CI 0.035–0.927, *P* = 0.042). However, the R^2^ for the association between the OS HR and the 1-year EFS was moderate (R^2^ = 0.348, 95% CI 0.00–0.759; *ρ* = 0.450, 95% CI -0.305–0.858, *P* = 0.22) (Table 1, Fig. 2b). The correlations between the OS HR and the 3-year EFS (R^2^ = 0.765, 95% CI 0.545–0.985; *ρ* = 0.717, 95% CI 0.10–0.936, *P* = 0.030) and the 5-year EFS (R^2^ = 0.695, 95% CI 0.423–0.967; *ρ* = 0.767, 95% CI 0.209–0.948, *P* = 0.016) were assessed as very good and good, respectively (Table 1, Fig. 2c-d).

**Table 1:** Correlations between surrogate endpoints and OS

Surrogate endpoint	R^**2**^ (95% CI)	ρ (95% CI)	*P-*value
EFS	0.747 (0.513–0.981)	0.683 (0.035–0.927)	0.042
1-year EFS	0.348 (0.00–0.759)	0.450 (−0.305–0.858)	0.22
3-year EFS	0.765 (0.545–0.985)	0.717 (0.10–0.936)	0.030
5-year EFS	0.695 (0.423–0.967)	0.767 (0.209–0.948)	0.016
1-year OS	0.089 (0.00–0.408)	0.214 (−0.642–0.833)	0.64
3-year OS	0.831 (0.650–1.00)	0.929 (0.584–0.990)	0.0025
5-year OS	0.809 (0.625–0.993)	0.767 (0.209–0.948)	0.016

Abbreviations: *CI* confidence interval; *OS* overall survival; *EFS* event-free survival

**Fig. 3:**
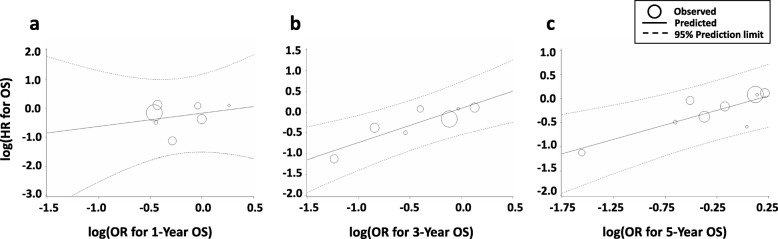
Correlation of OS HR with (**a**) 1-year OS, (**b**) 3-year OS, and (**c**) 5-year OS in Ewing sarcoma. HR, hazard ratio; OR, odds ratio; OS, overall survival

Similar to what we observed for the 1-year EFS, the correlation between the 1-year OS and the OS HR was poor (R^2^ = 0.089, 95% CI 0.00–0.408; *ρ* = 0.214, 95% CI -0.642–0.833, *P* = 0.64). Further, the 3-year OS (R^2^ = 0.831, 95% CI 0.650–1.00; *ρ* = 0.929, 95% CI 0.584–0.990, *P* = 0.0025) and the 5-year OS (R^2^ = 0.809, 95% CI 0.625–0.993; *ρ* = 0.767, 95% CI 0.209–0.948, *P* = 0.016) showed very good correlations with OS HR (Table 1, Fig. 3a-c).

**Fig. 4:**
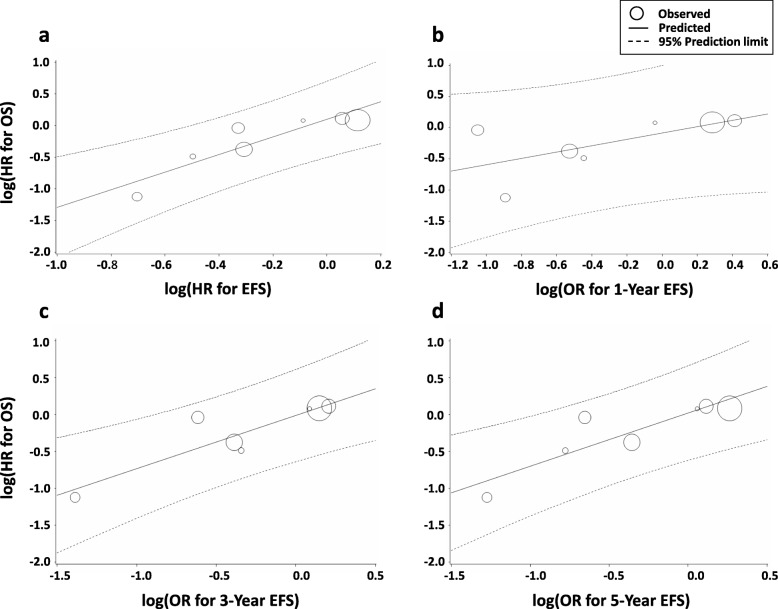
Correlation of EFS with OS for localized Ewing sarcoma. Correlation of (**a**) EFS HR, (**b**) 1-year EFS, (**c**) 3-year EFS, and (**d**) 5-year EFS. HR, hazard ratio; OR, odds ratio; OS, overall survival; EFS, event-free survival

**Fig. 5:**
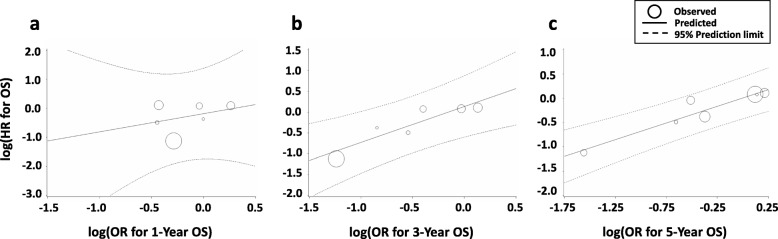
Correlation of (**a**) 1-year OS, (**b**) 3-year OS, and (**c**) 5-year OS with OS HR for localized Ewing sarcoma. HR, hazard ratio; OR, odds ratio; OS, overall survival

Furthermore, sensitivity analyses were conducted by removing the treatment arms of the metastatic and high-risk populations. There were 2 RCTs (INT-0091 and EICESS-92) in which metastatic ES was included. In INT-0091, all 120 patients in the metastatic subgroup had metastatic disease [9]. On the other hand, in EICESS-92, the definition of “high-risk” was a large localized tumor (≥100 ml) or metastatic disease [10]. Thus, the high-risk subgroup in EICESS-92 included 157 patients with metastatic disease and 335 patients with a non-metastatic large localized tumor. After the removal of these subgroups, localized ES analyses revealed an improved correlation between the intermediate endpoints and OS. The correlation between the EFS HR and the OS HR was very good (R^2^ = 0.818, 95% CI 0.625–1.00; *ρ* = 0.929, 95% CI 0.584–0.990, *P* = 0.0025) (Table 2, Fig. 4a). The R^2^ for the associations between the OS HR and the 1-year EFS remained moderate (R^2^ = 0.436, 95% CI 0.00–0.873; *ρ* = 0.750, 95% CI -0.007–0.961, *P* = 0.052) (Table 2, Fig. 4b). The correlations between the OS HR and the 3-year EFS (R^2^ = 0.807, 95% CI 0.604–1.00; *ρ* = 0.857, 95% CI 0.294–0.979, *P* = 0.014) and the 5-year EFS (R^2^ = 0.772, 95% CI 0.537–1.00; *ρ* = 0.929, 95% CI 0.584–0.990, *P* = 0.0025) were very good (Table 2, Fig. 4c-d). The correlation between the 1-year OS and the OS HR remained poor (R^2^ = 0.136, 95% CI 0.00–0.535; *ρ* = 0.257, 95% CI -0.701–0.884, *P* = 0.62); however, the 3-year OS (R^2^ = 0.858, 95% CI 0.693–1.00; *ρ* = 0.943, 95% CI 0.559–0.994, *P* = 0.0048) and the 5-year OS (R^2^ = 0.895, 95% CI 0.778–1.00; *ρ* = 0.929, 95% CI 0.584–0.990, *P* = 0.0025) showed nearly excellent correlations with OS HR (Table 2, Fig. 5a-c).

**Table 2:** Correlations between surrogate endpoints and OS in localized Ewing sarcoma

Surrogate endpoint	R^2^ (95% CI)	ρ (95% CI)	*P-*value
EFS	0.818 (0.625–1.00)	0.929 (0.584–0.990)	0.0025
1-year EFS	0.436 (0.00–0.873)	0.750 (−0.007–0.961)	0.052
3-year EFS	0.807 (0.604–1.00)	0.857 (0.294–0.979)	0.014
5-year EFS	0.772 (0.537–1.00)	0.929 (0.584–0.990)	0.0025
1-year OS	0.136 (0.00–0.535)	0.257 (−0.701–0.884)	0.62
3-year OS	0.858 (0.693–1.00)	0.943 (0.559–0.994)	0.0048
5-year OS	0.895 (0.778–1.00)	0.929 (0.584–0.990)	0.0025

Abbreviations: *CI* confidence interval; *OS* overall survival; *EFS* event-free survival

In the present study, 2 RCTs were conducted in the 1970s when CT had not been introduced in clinical practice; thus, the staging or evaluation of lung metastasis might be more difficult in these RCTs than in more recent trials. Therefore, we conducted further sensitivity analyses by excluding these 2 RCTs. The results demonstrated that the correlation between HRs for EFS and OS was good, with R^2^ = 0.519 (95% CI 0.041–0.997) and *ρ* = 0.800 (95% CI -0.280–0.986).

## Discussion

In RCTs of ES, EFS is often selected as the primary endpoint. The advantages of EFS over OS as an endpoint of RCTs are that EFS can be available in a shorter period with a smaller sample size, and subsequently at a lower cost than OS, and that post-progression treatments do not influence EFS. Furthermore, EFS is an important measure that indicates how long the patient remains disease-free in an adjuvant treatment trial, and it is considered to be a beneficial outcome, especially in the study of pediatric cancer therapy. On the other hand, the disadvantages of EFS are that EFS can be affected by the frequency and subjectivity of the surveillance of events, thus including some ambiguity, and that EFS does not always correlate with OS. It has been debated whether EFS is an actual endpoint in its own right or is simply a surrogate for OS. However, the correlation between EFS and OS has never been verified in ES trials. In this study, data from RCTs of systemic chemotherapy for ES were collected, and the correlation of EFS and OS was analyzed using a meta-analytic approach. We identified only 7 relevant trials, reflecting the rare occurrence of the disease. This is the first study to analyze the relationship between EFS and OS in RCTs of ES.

The correlation between the EFS and OS HR in RCTs of newly diagnosed ES was assessed as good, with R^2^ = 0.747 and *ρ* = 0.683. Several RCTs used in this analysis included metastatic disease, and not all enrolled patients had localized ES. Although both metastatic and localized ES have the same primary treatment strategy, the associated prognoses are quite different, making it difficult to analyze them together. Therefore, sensitivity analyses were performed with only the treatment arms for cases with localized disease, excluding metastatic and high-risk disease cases, and the correlation between EFS and OS showed an R^2^ of 0.818 and ρ of 0.929, indicating a very good correlation. The same trend was confirmed by sensitivity analyses of the other time-to-event endpoints, i.e., 1- to 5-year EFS and 1- to 5-year OS. Our results suggest that EFS has the potential to be used as a surrogate for OS in RCTs of newly diagnosed and localized ES. In the sensitivity analysis, the study arms that included metastatic disease were excluded. EFS HR and OS HR of the metastatic disease group in the excluded INT-0091 trial were 0.95 (95% CI 0.63–1.43) and 0.56 (95% CI 0.36–0.87), respectively. However, in the localized disease group, the hazard ratios of EFS and OS between the two arms were 0.72 (95% CI 0.57–0.91) and 0.96 (95% CI 0.68–1.35), respectively. These data revealed the discrepancy between the significantly better OS in the experimental treatment arm and the similar EFS in both arms of the metastatic disease group of the INT-0091 trial. On the other hand, such discrepancy was not observed in another study that was excluded; EICESS-92. In the high-risk group in the EICESS-92 trial, the hazard ratios of EFS and OS between the two arms were 0.83 (95% CI 0.65–1.05) and 0.85 (95% CI 0.66–1.10), respectively. Thus, the exclusion of the metastatic group of the INT-0091 trial would lead to a better correlation between EFS HR and OS HR in localized ES. The reason for the discrepancy between EFS HR and OS HR in the INT-0091 trial was unknown. This needs to be explained by analyzing individual patient data.

The average median follow-up time of the included RCTs was 6.79 years. The correlation between 3-year EFS and OS (R^2^ = 0.765, *ρ* = 0.717) was comparable to that of the EFS HR and OS, and analyses of the 3-year EFS restricted to localized disease cases further showed a very good correlation with OS (R^2^ = 0.807, *ρ* = 0.857). When EFS is selected as the primary endpoint in RCTs of ES, it is considered necessary to conduct follow-up for at least 3 years. In one eligible RCT, 3-year EFS was the primary endpoint [10]. In the European Intergroup Cooperative Ewing’s Sarcoma Study-92 trial, the high- and low-risk subgroups included 157 and 4 patients with metastatic disease, respectively. Because the correlation of OS with 3-year EFS tended to be more favorable when examining localized ES, if 3-year EFS is chosen as the primary endpoint, studies limited to localized ES cases may be preferable.

In localized lung cancer, a re-analysis of six meta-analyses involving 60 RCTs with 15,071 patients demonstrated that the strength of the correlation between disease-free survival (DFS) and OS in RCTs of adjuvant chemotherapy was R^2^ = 0.92, which was excellent. For operable lung cancer, DFS is a reliable surrogate endpoint in RCTs performed in adjuvant settings [14]. In RCTs of resectable gastric cancer, the correlation between DFS and OS is also very good (R^2^ = 0.964), and DFS was concluded to be an acceptable surrogate for OS [15]. On the other hand, the evaluation of the surrogacy of DFS for OS in 22 RCTs of gastro-esophageal cancer demonstrated that DFS did not correlate with OS (R^2^ = 0.27) [16]. In an analysis of 6 RCTs of adjuvant chemotherapy, which included 12,676 cases of localized colon cancer, the overall correlation between DFS and OS was modest to poor (R^2^ = 0.37), and DFS was not a good surrogate for OS in RCTs of stage II colon cancer [17]. Furthermore, in the field of sarcoma, the surrogacy of progression-free survival (PFS) for OS was also investigated using RCTs of advanced soft tissue sarcomas. The correlation between PFS and OS was modest at best; thus, the surrogacy of PFS could not be confirmed in the analyses [18, 19]. These observations suggest that confirmation of the correlation between surrogate endpoints and OS is important.

Response to chemotherapy may be the surrogate endpoint for which results are obtained most quickly. In RCTs of osteosarcoma, the treatment strategy for changing the postoperative regimen is widely based on the histological response to preoperative chemotherapy [20, 21]. Conversely, none of the ES trials selected histological response as an endpoint. Histological response is a significant predictor of favorable outcomes in ES [22]. Nevertheless, only two studies examined histological responses using Huvos grading criteria in the excised tumor tissue [10, 13]. Because the strategy of changing the postoperative regimen based on histological response has not been established for ES [2], histological response may not necessarily be investigated in ES trials.

Our study had some limitations. 1) The study was based on published, and not individual data. 2) Only 7 RCTs of ES were eligible; thus, the number of RCTs evaluated in the surrogacy analysis was minimal. 3) Definitions and start dates of the time-to-event endpoints varied across trials. 4) The study phase was not clarified in any of the RCTs, and there was no description of post-protocol treatments. An ITT analysis description was observed in only 2 studies, suggesting that the quality of the studies was not excellent. 5) Metastatic disease was included in several trials, and not all studies focused purely on localized ES. In total, 281 (7.8%) of 3612 patients had metastatic disease. This limits the ability to conclude from the study results. 6) The study period for each RCT was from 1973 to 2010. This might increase the heterogeneity among the RCTs included in this study.

## Conclusions

As a surrogate endpoint in RCTs of newly diagnosed ES, EFS showed a good correlation with OS, and the correlation was stronger in cases of localized disease. EFS may be a useful endpoint in RCTs of ES; this is worth verifying using individual patient data in the future.

## Supplementary information

**Additional file 1: Table S1.** Detailed description of RCTs. RCT, randomized controlled trial; ITT, intention-to-treat; OS, overall survival; EFS, event-free survival; DFS, disease-free survival; VCR, vincristine; ActD, actinomycin D; CPA, cyclophosphamide; ADM (DOX), doxorubicin; IFM, ifosfamide; ETP, etoposide; VAC, VCR + ActD+CPA; VACA, VCR + ActD+CPA + ADM; VAIA, VCR + ActD+IFM + ADM; VAI, VCR + ActD+IFM; VDC, VCR + doxorubicin+CPA; IE, IFM + ETP; EVAIA, ETP + VCR + ActD+IFM + ADM.

**Additional file 2: Figure S1.** Forest plot of EFS with standard versus experimental chemotherapy. CI, confidence interval; EFS, event-free survival; HR, high risk; IV, inverse variance; Meta, metastatic disease; N-meta, non-metastatic disease; SE, standard error; SR, standard risk.

**Additional file 3: Figure S2.** Forest plot of OS with standard versus experimental chemotherapy. CI, confidence interval; HR, high risk; IV, inverse variance; Meta, metastatic disease; N-meta, non-metastatic disease; OS, overall survival; SE, standard error; SR, standard risk.

## Data Availability

The datasets generated and analyzed in the current study are available from the corresponding author on reasonable request.

## Abbreviations

ES: Ewing sarcoma
RCT: Randomized controlled trial
OS: Overall survival
EFS: Event-free survival
ITT: Intention-to-treat
AE: Adverse event
HR: Hazard ratio
CI: Confidence interval
OR: Odds ratio
DFS: Disease-free survival
