# Supplementary material for: A meta-analytic evaluation of the correlation between event-free survival and overall survival in randomized controlled trials of newly diagnosed Ewing sarcoma
Source: BMC Cancer. 2020 May 5;20:379. doi: 10.1186/s12885-020-06871-9 (PMC7201711; doi:10.1186/s12885-020-06871-9)
Supplement: Supplementary file 1 — Additional file 1: Table S1. Detailed description of RCTs. RCT, randomized controlled trial; ITT, intention-to-treat; OS, overall survival; EFS, event-free survival; DFS, disease-free survival; VCR, vincristine; ActD, actinomycin D; CPA, cyclophosphamide; ADM (DOX), doxorubicin; IFM, ifosfamide; ETP, etoposide; VAC, VCR + ActD+CPA; VACA, VCR + ActD+CPA + ADM; VAIA, VCR + ActD+IFM + ADM; VAI, VCR + ActD+IFM; VDC, VCR + doxorubicin+CPA; IE, IFM + ETP; EVAIA, ETP + VCR + ActD+IFM + ADM. [file 12885_2020_6871_MOESM1_ESM.zip › Additional_File1contR2.docx]

**Additional File 1: Table S1 (Online only) (continued).**

**Table S1 (continued). Detailed description of RCTs**

| **Author and year of publication** | **Standard regimen dose (per cycle)** | **Experimental regimen** | **Experimental regimen dose (per cycle)** | **Study phase** | **Primary endpoint** | **Other specified endpoint** | **ITT analysis** | **Post-protocol treatment** |
| --- | --- | --- | --- | --- | --- | --- | --- | --- |
| Nesbit 1990 | VCR 1.5 mg/m^2^ weekly ActD 15 µg/kg/day ×5 days CPA 500 mg/m^2^ weekly | 1) VACA | VCR 1.5 mg/m^2^ weekly ActD 15 µg/kg/day x5days CPA 500 mg/m^2^ weekly ADM 60 mg/m^2^ | not specified | OS and time to relapse | not specified | not specified | not specified |
|  |  | 2) VAC+ Lung irradiation | VCR 1.5 mg/m^2^ weekly  ActD 15 µg/kg/day ×5 days  CPA 500 mg/m^2^ weekly  Lung irradiation 15-18 Gy |  |  |  |  |  |
| Burgert 1990 | VCR 1.5 mg/m^2^ ActD 0.45 mg/m^2^ CPA 500 mg/m^2^ ADM 60 mg/m^2^ | VACA high dose | VCR 1.5 mg/m^2^ ActD 0.45 mg/m^2^ CPA 1400 mg/m^2^ ADM 75 mg/m^2^ | not specified | OS and time to relapse | DFS | not specified | not specified |
| Grier 2003 | VCR 2 mg/m^2^ ActD 1.25 mg/m^2^2 CPA 1200 mg/m^2^ ADM 75 mg/m^2^ | VACA+IE | VCR 2 mg/m^2^ ActD 1.25 mg/m^2^2 CPA 1200 mg/m^2^ ADM 75 mg/m^2^ IFM 1800 mg/m^2^/day ×5 days ETP 100 mg/m^2^/day ×5 days | not specified | EFS | not specified | not specified | not specified |
| Paulussen 2008 | VCR 1.5 mg/m^2^ ActD 0.5 mg/m^2^/day ×3 days IFM 2000 mg/m^2^/day ×3 days ADM 30 mg/m^2^/day ×2 days | VACA | VCR 1.5 mg/m^2^ ActD 0.5 mg/m^2^/day ×3 days CPA 1200 mg/m^2^ ADM 30 mg/m^2^/day ×2 days | not specified | 3-year EFS | OS, toxicity | Yes | not specified |
|  |  | EVAIA | ETP 150 mg/m^2^/day ×3 days VCR 1.5 mg/m^2^ ActD 0.5 mg/m^2^/day ×3 days IFM 2000 mg/m^2^/day ×3 days ADM 30 mg/m^2^/day ×2 days |  |  |  |  |  |
| Granowetter 2009 | VCR 1.5 mg/m^2^ DOX 75 mg/m^3^ CPA 1200 mg/m^2^ IFM 1800 mg/m^2^/day ×5 days ETP 100 mg/m^2^/day ×5 days | VDC+IE high dose | VCR 1.5 mg/m^2^ DOX 75 mg/m^3^ CPA 2100 mg/m^2^/day ×2 days IFM 2400 mg/m^2^/day ×5 days ETP 100 mg/m^2^/day ×5 days | not specified | EFS | not specified | not specified | not specified |
| Womer 2012 | VCR 2 mg/m^2^ DOX 37.5 mg/m^3^/day ×2 days CPA 1200 mg/m^2^ IFM 1800 mg/m^2^/day ×5 days ETP 100 mg/m^2^/day ×5 days | VDC+IE 2-week interval | VCR 2 mg/m^2^ DOX 37.5 mg/m^3^/day ×2 days CPA 1200 mg/m^2^ IFM 1800 mg/m^2^/day ×5 days ETP 100 mg/m^2^/day ×5 days | not specified | EFS | not specified | not specified | not specified |
| Le Deley 2014 | VCR 1.5 mg/m^2^ ActD 0.75 mg/m^2^/day ×2 days IFM 3000 mg/m^2^/day ×2 days | VAC | VCR 1.5 mg/m^2^ ActD 0.75 mg/m^2^/day ×2 days CPA 1500 mg/m^2^ | not specified | EFS | OS | Yes | not specified |
