# Supplementary material for: A meta-analytic evaluation of the correlation between event-free survival and overall survival in randomized controlled trials of newly diagnosed Ewing sarcoma
Source: BMC Cancer. 2020 May 5;20:379. doi: 10.1186/s12885-020-06871-9 (PMC7201711; doi:10.1186/s12885-020-06871-9)
Supplement: Supplementary file 1 — Additional file 1: Table S1. Detailed description of RCTs. RCT, randomized controlled trial; ITT, intention-to-treat; OS, overall survival; EFS, event-free survival; DFS, disease-free survival; VCR, vincristine; ActD, actinomycin D; CPA, cyclophosphamide; ADM (DOX), doxorubicin; IFM, ifosfamide; ETP, etoposide; VAC, VCR + ActD+CPA; VACA, VCR + ActD+CPA + ADM; VAIA, VCR + ActD+IFM + ADM; VAI, VCR + ActD+IFM; VDC, VCR + doxorubicin+CPA; IE, IFM + ETP; EVAIA, ETP + VCR + ActD+IFM + ADM. [file 12885_2020_6871_MOESM1_ESM.zip › Additional_File1R2.docx]

**Additional File 1: Table S1 (Online only).**

**Table S1. Detailed description of RCTs**

| **Author and year of publication** | **Study name** | **Study period** | **Median follow-up** | **No. of patients in standard arm** | **No. of patients in experimental arm** | **Total no. of patients** | **No. of patients with metastatic disease** | **Standard regimen** |
| --- | --- | --- | --- | --- | --- | --- | --- | --- |
| Nesbit 1990 | IESS-I | May 1973 - Nov 1978 | 6 years | 74 | 1) 148  2) 109 | 331 | 0 | VAC |
| Burgert 1990 | IESS-II | Nov 1978 - Dec 1982 | 5.6 years | 106 | 108 | 214 | 0 | VACA moderate dose |
| Grier 2003 | INT-0091 non metastatic | Dec 1988 -  Nov 1992 | 8.1 years | 200 | 198 | 398 | 0 | VACA |
|  | INT-0091 metastatic |  |  | 62 | 58 | 120 | 120 |  |
| Paulussen 2008 | EICESS-92 Standard Risk | Jul 1992 -  Dec 1999 | 8.5 years | 76 | 79 | 155 | 4 | VAIA |
|  | EICESS-92 High Risk |  |  | 240 | 252 | 492 | 157 |  |
| Granowetter 2009 | INT-0154 | May 1995 - Sep 1998 | 8.3 years | 231 | 247 | 478 | 0 | VDC+IE standard dose |
| Womer 2012 | AEWS0031 | May 2001 - Aug 2005 | 5.1 years | 284 | 284 | 568 | 0 | VDC+IE 3-week interval |
| Le Deley 2014 | Euro-EWING99- R1 | Feb 2000 - Mar 2010 | 5.9 years | 425 | 431 | 856 | 0 | VAI |

Abbreviations: RCT, randomized controlled trial; ITT, intention-to-treat; OS, overall survival; EFS, event-free survival; DFS, disease-free survival; VCR, vincristine; ActD, actinomycin D; CPA, cyclophosphamide; ADM (DOX), doxorubicin; IFM, ifosfamide; ETP, etoposide; VAC, VCR+ActD+CPA;

VACA, VCR+ActD+CPA +ADM; VAIA, VCR+ActD+IFM+ADM; VAI, VCR+ActD+IFM; VDC, VCR+doxorubicin+CPA; IE, IFM+ETP; EVAIA, ETP+VCR+ActD+IFM+ADM.
