# Supplementary material for: A meta-analytic evaluation of the correlation between event-free survival and overall survival in randomized controlled trials of newly diagnosed Ewing sarcoma
Source: BMC Cancer. 2020 May 5;20:379. doi: 10.1186/s12885-020-06871-9 (PMC7201711; doi:10.1186/s12885-020-06871-9)
Supplement: Supplementary file 2 — Additional file 2: Figure S1. Forest plot of EFS with standard versus experimental chemotherapy. CI, confidence interval; EFS, event-free survival; HR, high risk; IV, inverse variance; Meta, metastatic disease; N-meta, non-metastatic disease; SE, standard error; SR, standard risk. [file 12885_2020_6871_MOESM2_ESM.pptx]

## Slide 1
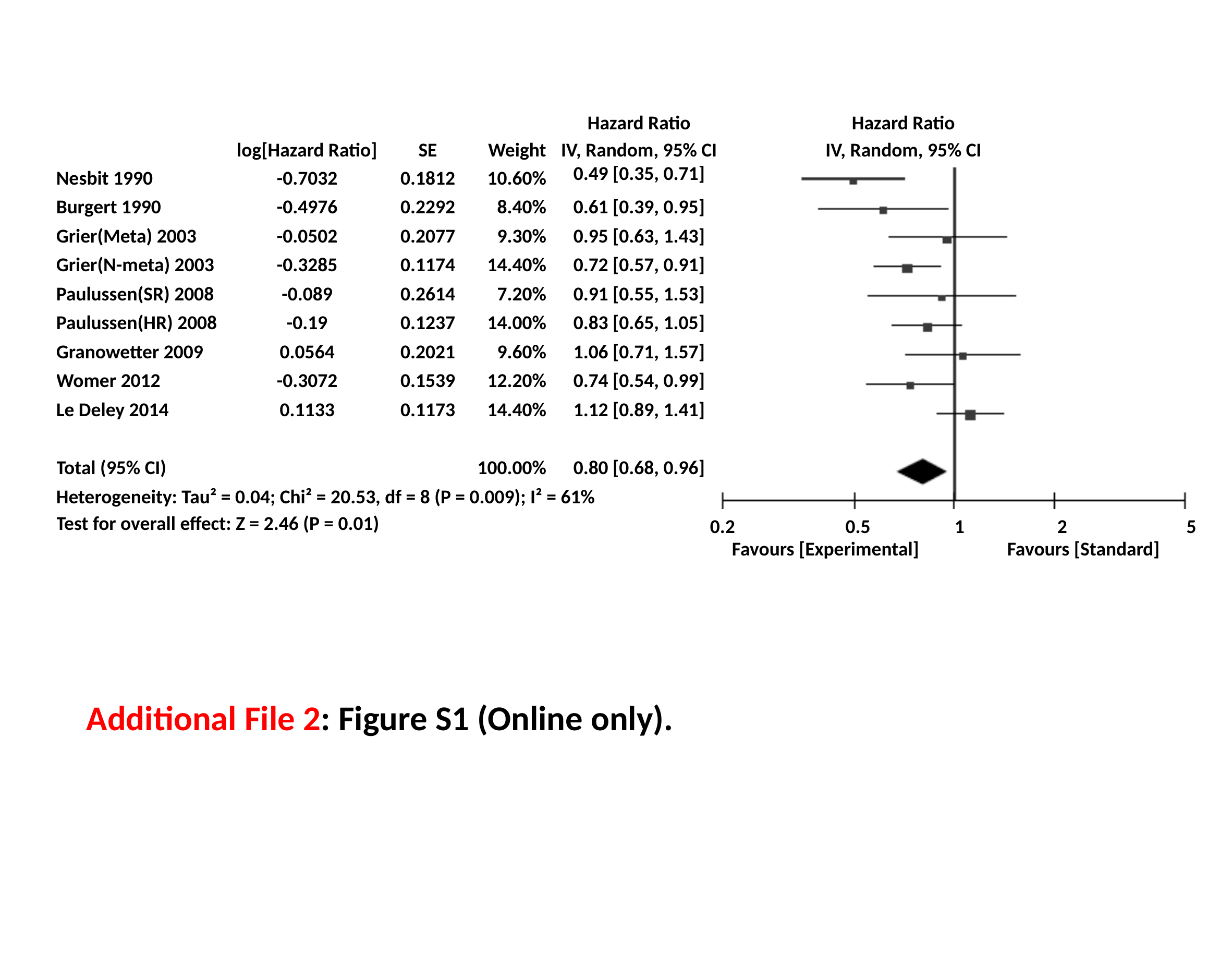

| | | | | | | | Hazard Ratio | | | | Hazard Ratio | | | |
| --- | --- | --- | --- | --- | --- | --- | --- | --- | --- | --- | --- | --- | --- | --- |
| | log[Hazard Ratio] | log[Hazard Ratio] | SE | SE | SE | Weight | IV, Random, 95% CI | | | | IV, Random, 95% CI | | | |
| Nesbit 1990 | -0.7032 | -0.7032 | 0.1812 | 0.1812 | 0.1812 | 10.60% | 0.49 [0.35, 0.71] | | | | | | | |
| Burgert 1990 | -0.4976 | -0.4976 | 0.2292 | 0.2292 | 0.2292 | 8.40% | 0.61 [0.39, 0.95] | | | | | | | |
| Grier(Meta) 2003 | -0.0502 | -0.0502 | 0.2077 | 0.2077 | 0.2077 | 9.30% | 0.95 [0.63, 1.43] | | | | | | | |
| Grier(N-meta) 2003 | -0.3285 | -0.3285 | 0.1174 | 0.1174 | 0.1174 | 14.40% | 0.72 [0.57, 0.91] | | | | | | | |
| Paulussen(SR) 2008 | -0.089 | -0.089 | 0.2614 | 0.2614 | 0.2614 | 7.20% | 0.91 [0.55, 1.53] | | | | | | | |
| Paulussen(HR) 2008 | -0.19 | -0.19 | 0.1237 | 0.1237 | 0.1237 | 14.00% | 0.83 [0.65, 1.05] | | | | | | | |
| Granowetter 2009 | 0.0564 | 0.0564 | 0.2021 | 0.2021 | 0.2021 | 9.60% | 1.06 [0.71, 1.57] | | | | | | | |
| Womer 2012 | -0.3072 | -0.3072 | 0.1539 | 0.1539 | 0.1539 | 12.20% | 0.74 [0.54, 0.99] | | | | | | | |
| Le Deley 2014 | 0.1133 | 0.1133 | 0.1173 | 0.1173 | 0.1173 | 14.40% | 1.12 [0.89, 1.41] | | | | | | | |
| | | | | | | | | | | | | | | |
| Total (95% CI) | | | | | | 100.00% | 0.80 [0.68, 0.96] | | | | | | | |
| Heterogeneity: Tau² = 0.04; Chi² = 20.53, df = 8 (P = 0.009); I² = 61% | | | | | | | | | | | | | | |
| Test for overall effect: Z = 2.46 (P = 0.01) | | | | | | | | | | | | | | |
| | | | | | | | | Favours [Experimental] | | | | Favours [Standard] | | |
0.2 0.5 1 2 5
Additional File 2: Figure S1 (Online only).
