# Supplementary material for: A meta-analytic evaluation of the correlation between event-free survival and overall survival in randomized controlled trials of newly diagnosed Ewing sarcoma
Source: BMC Cancer. 2020 May 5;20:379. doi: 10.1186/s12885-020-06871-9 (PMC7201711; doi:10.1186/s12885-020-06871-9)
Supplement: Supplementary file 3 — Additional file 3: Figure S2. Forest plot of OS with standard versus experimental chemotherapy. CI, confidence interval; HR, high risk; IV, inverse variance; Meta, metastatic disease; N-meta, non-metastatic disease; OS, overall survival; SE, standard error; SR, standard risk. [file 12885_2020_6871_MOESM3_ESM.pptx]

## Slide 1
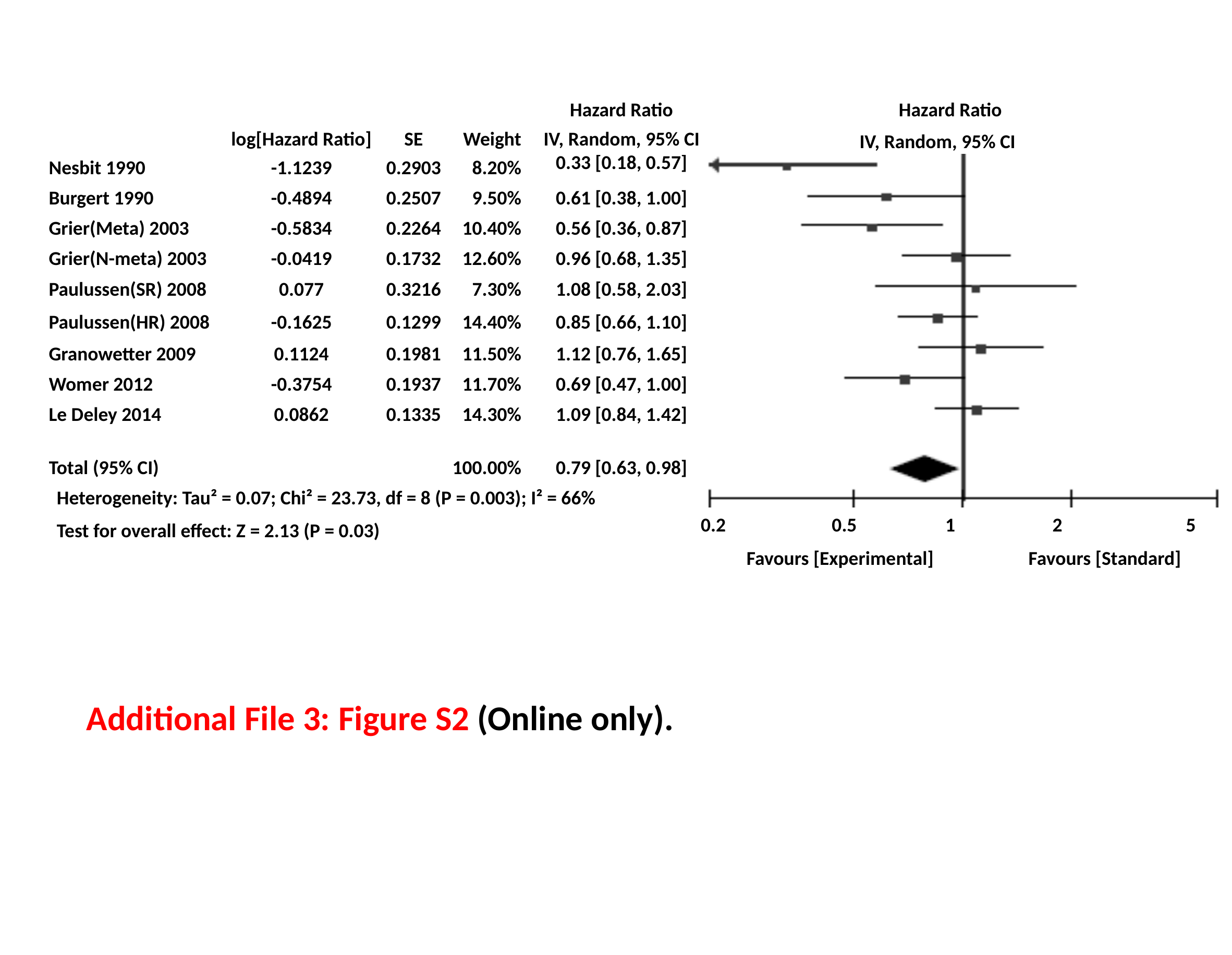

| | | | | | | | Hazard Ratio | | | Hazard Ratio | | | | |
| --- | --- | --- | --- | --- | --- | --- | --- | --- | --- | --- | --- | --- | --- | --- |
| | log[Hazard Ratio] | log[Hazard Ratio] | SE | SE | Weight | Weight | IV, Random, 95% CI | | | IV, Random, 95% CI | | | | |
| Nesbit 1990 | -1.1239 | -1.1239 | 0.2903 | 0.2903 | 8.20% | 8.20% | 0.33 [0.18, 0.57] | | | | | | | |
| Burgert 1990 | -0.4894 | -0.4894 | 0.2507 | 0.2507 | 9.50% | 9.50% | 0.61 [0.38, 1.00] | | | | | | | |
| Grier(Meta) 2003 | -0.5834 | -0.5834 | 0.2264 | 0.2264 | 10.40% | 10.40% | 0.56 [0.36, 0.87] | | | | | | | |
| Grier(N-meta) 2003 | -0.0419 | -0.0419 | 0.1732 | 0.1732 | 12.60% | 12.60% | 0.96 [0.68, 1.35] | | | | | | | |
| Paulussen(SR) 2008 | 0.077 | 0.077 | 0.3216 | 0.3216 | 7.30% | 7.30% | 1.08 [0.58, 2.03] | | | | | | | |
| Paulussen(HR) 2008 | -0.1625 | -0.1625 | 0.1299 | 0.1299 | 14.40% | 14.40% | 0.85 [0.66, 1.10] | | | | | | | |
| Granowetter 2009 | 0.1124 | 0.1124 | 0.1981 | 0.1981 | 11.50% | 11.50% | 1.12 [0.76, 1.65] | | | | | | | |
| Womer 2012 | -0.3754 | -0.3754 | 0.1937 | 0.1937 | 11.70% | 11.70% | 0.69 [0.47, 1.00] | | | | | | | |
| Le Deley 2014 | 0.0862 | 0.0862 | 0.1335 | 0.1335 | 14.30% | 14.30% | 1.09 [0.84, 1.42] | | | | | | | |
| | | | | | | | | | | | | | | |
| Total (95% CI) | | | | | 100.00% | 100.00% | 0.79 [0.63, 0.98] | | | | | | | |
| Heterogeneity: Tau² = 0.07; Chi² = 23.73, df = 8 (P = 0.003); I² = 66% | | | | | | | | | | | | | | |
| Test for overall effect: Z = 2.13 (P = 0.03) | | | | | | | | | | | | | | |
| | | | | | | | | | Favours [Experimental] | | | Favours [Standard] | | |
0.2 0.5 1 2 5
Additional File 3: Figure S2 (Online only).
